# A case of percutaneous tricuspid valve infective endocarditis vegetation debulking using the AngioJet rheolytic catheter system—A novel therapeutic use

**DOI:** 10.1002/ccr3.4314

**Published:** 2021-06-24

**Authors:** Liam Marsden Back, Siobhan Hurley, Justin Beardsley, Virag Kushwaha

**Affiliations:** ^1^ Prince of Wales Hospital Randwick NSW Australia; ^2^ The University of New South Wales Sydney NSW Australia; ^3^ Marie Bahir Institute The University of Sydney Camperdown NSW Australia

**Keywords:** AngioJet rheolytic catheter, debulking, tricuspid valve endocarditis

## Abstract

In patients with fulminant tricuspid valve infective endocarditis precluded from cardiothoracic intervention based on comorbidities or clinical status, percutaneous vegetation debulking utilizing the AngioJet rheolytic catheter system appears a viable rescue option to achieve source control.

## Graphical abstract

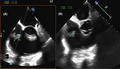

## 1 INTRODUCTION

Tricuspid valve infective endocarditis (IE) has the potential to lead to overwhelming sepsis despite optimal medical therapy, particularly in the presence of large vegetations. A subset of patients with this disease are precluded from cardiothoracic intervention for source control due to their high operative risk and are candidates for percutaneous debulking procedures. Several centers have had experience with the AngioVac (Angiodynamics, Inc) aspiration system with described success. The use of the AngioJet (Possis Medical, Inc) rheolytic catheter system, although not previously described, appeared a viable rescue option in our patient. The procedure resulted in an immediate echocardiographic improvement in the vegetation's appearance, as well as biochemical and clinical improvement to our patient's condition. Our patient was discharged home well and has remained stable and free from recurrence of bacteremia. This case highlights a novel therapeutic use of the AngioJet rheolytic catheter as a means of source control in tricuspid valve IE in patients without a surgical option.

## 2 CASE PRESENTATION

A 52‐year‐old man presented to our Emergency Department with a 5‐day history of subjective fevers, rigors, cough, neck pain, and widespread myalgia. His past history was notable for eczema, asthma, hypertension, hypercholesterolemia and a history of a L5/S1 spinal fixation in the setting of a work‐related injury. He had no cardiac devices, prosthetic valves or joints, or other metalware. His social history was significant for alcohol misuse consuming up to 10 standard drinks per day, and he had no history of intravenous drug use. On initial examination, he was febrile to 38.2°C and hemodynamically stable. His cardiovascular, respiratory, and abdominal examination were unremarkable. There were no peripheral stigmata of IE.

Initial investigations revealed raised inflammatory markers with a white cell count of 12 × 10^9^/L and c‐reactive protein level of 243 mg/L. He had a thrombocytopenia with platelets of 52 × 10^9^/L and mildly deranged liver function. His chest X‐ray showed clear lung fields. Following admission, his symptoms rapidly progressed with worsening neck pain and mild left upper and lower limb weakness on examination. An urgent brain and spinal magnetic resonance imaging (MRI) revealed a prevertebral phlegmon extending from the skull to C4/C5. These images were reviewed by our neurosurgical team who felt that no surgical intervention was indicated.

His admission blood cultures returned positive for gram‐positive cocci, and he was empirically commenced on intravenous (IV) flucloxacillin 2 g every 6 hours and vancomycin on day 2 of his admission. This was narrowed to vancomycin monotherapy once sensitivities returned demonstrating methicillin‐resistant *S aureus* (MRSA). Initial transthoracic echocardiography (TTE) demonstrated normal valvular function with no evidence of vegetation.

He promptly developed type two respiratory failure requiring high‐dependency unit transfer for noninvasive ventilation. A computed tomography (CT) scan of his chest showed scattered ground‐glass and nodular opacities suggestive of septic emboli.

A repeat TTE 6 days into admission now demonstrated a mobile echodensity on the anterior tricuspid valve leaflet with mild tricuspid regurgitation. Clinically, he remained febrile and his blood cultures failed to clear despite therapeutic vancomycin with levels consistently between 20‐25 mcg/mL. Vancomycin susceptibility testing was performed on his day 9 isolate, and the minimal inhibitory concentration was 1 mg/L, suggesting that isolate was sensitive to vancomycin. By day 11, he deteriorated further from a respiratory perspective and oral sulfamethoxazole‐trimethoprim 800/160 mg twice daily was added to his therapy before being escalated to IV linezolid 600 mg twice daily on day 14. Cardiothoracic, neurosurgery and ear, nose, and throat teams all felt he was not a surgical candidate given his medical comorbidities and concurrent sepsis.

By day 17 of his admission, he had developed an acute kidney injury with creatinine of 126 μmol/L. He remained febrile, with raised inflammatory markers and a persistent MRSA bacteremia. Transoesophageal echocardiography (TOE) demonstrated an enlarging tricuspid valve vegetation, now measuring 2.1 × 2.6 cm (video S1). Moderate tricuspid regurgitation and pulmonary hypertension were also noted. Cardiothoracic opinion was once again sought, again with the consensus that there was no safe surgical treatment option. His antimicrobials subsequently were changed to salvage therapy with IV daptomycin 1.2 g daily and IV ceftaroline 600 mg three times daily.

An urgent multidisciplinary review reached a consensus to proceed with a percutaneous debulking with approval from the hospital executive under compassionate grounds; a procedure not performed locally previously. The AngioVac system that has previously been described for percutaneous vegetectomy was not available in our center.

**FIGURE 1:**
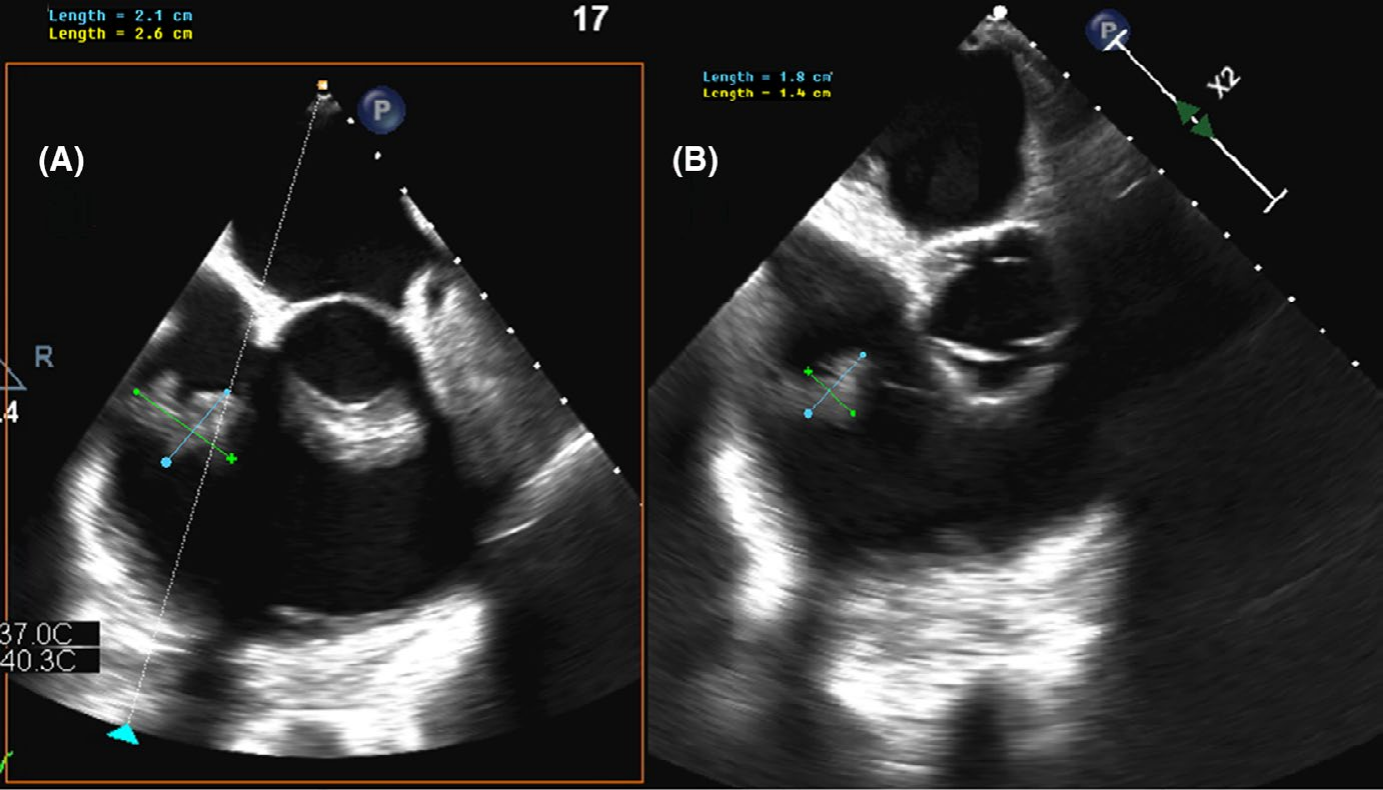
Mid‐esophageal short‐axis transoesophageal echocardiogram images demonstrating the tricuspid valve vegetation pre A, and post B, AngioJet debulking, with a greater than 50% reduction in size

The procedure was planned for sequential attempts at debulking, first with available percutaneous snare devices, and if unsuccessful, to proceed with rheolytic therapy with a 5F AngioJet catheter. Our patient was anesthetized with general anesthetic and had a Philips X8‐2t 3D (Koninklijke Phillips) TOE probe inserted into the esophagus and stomach to guide the procedure. Femoral venous access was obtained using ultrasound guidance, and a 14F venous sheath inserted. An 8.5F Agilis (medium curve) steerable catheter was then positioned in the right atrium above the tricuspid valve, with the tip deflected toward the anterior leaflet of the tricuspid valve. Multiple attempts at snaring the vegetation were made with both a 30 mm Ensnare and 25 mm Amplatzer Gooseneck snare. Due to the broad‐based nature of the vegetation however, it was difficult to catch at the base of the vegetation without pulling firmly on valvular and subvalvular structures. We subsequently proceeded to insert a 5F Angiojet aspiration catheter through the 8.5F Agilis sheath with multiple passes across the vegetation (Video S2, Video S3). Passes were timed for approximately 15 seconds individually for a total time of 120 seconds, mindful of hemolytic complications. The AngioJet catheter was advanced approximately 10 cm from the lumen of the Agilis catheter with guidance toward the vegetation by simultaneous TOE imaging. The procedure was completed without immediate hemodynamic or respiratory instability. Repeat TOE postprocedure demonstrated a greater than 50% reduction in the size of the vegetation (Figure 1), as well as a marked clinical improvement with down trending inflammatory markers, clearance of blood cultures, and resolution of fevers over the next 24 hours.

His postprocedural course was complicated by oliguric renal failure requiring hemodialysis, which was successfully weaned after 4 weeks. It is unclear to what degree the procedure itself contributed to the development of renal failure, as hemolysis resulting in kidney injury is a known complication of using the AngioJet system in large vessels for prolonged periods. Despite multiple specialty teams' assessment, it was never apparent which of our patient's secondarily seeded infective sites was most important; however, his neurology, respiratory support, and osteoarthritic pain improved completely at time of discharge. In total, he received 2 months of IV therapy before being stepped down to oral sulfamethoxazole‐trimethoprim 800/160 mg twice daily. TTE at this time demonstrated normal left ventricular size with hyperdynamic systolic function, normal right ventricular size and systolic function, severe tricuspid regurgitation, and mild pulmonary hypertension. Following rehabilitation, he was discharged home fully independent, with normal renal function after 12 weeks, and plans to continue oral antibiotics pending outpatient review in 6 weeks.

## 3 DISCUSSION

Infective endocarditis remains a significant disease with a 1‐year mortality rate of 30%, with little improvement to this figure over the past three decades despite an increased understanding of the pathological drivers and treatment modalities available.^1^ Right‐sided native valve IE, although accounting for only 5%‐10% of IE cases,^2^ continues to prove a management challenge for cardiologists, infectious disease physicians, and cardiothoracic surgeons. Staphylococcus aureus is the predominant causative organism in right‐sided IE, attributable for up to 90% of cases, with a slowly increasing contribution of MRSA species and polymicrobial infections.^3^

Intravenous antibiotics directed toward an identified organism for 2‐6 weeks remain the foundation of management for all right‐sided IE.^4^ More fulminant disease courses are not uncommon however, with surgical management considered in several circumstances such as persistent bacteremia despite adequate medical therapy, recurrent pulmonary emboli, tricuspid annular abscesses, and right heart failure with severe tricuspid regurgitation.^5^ Rates of surgical intervention in patients with right‐sided IE have been estimated to be in the range of 5%‐40%, and the indications are far less well‐defined than those for left heart lesions.^6^ Surgical management has typically consisted of radical vegetectomy, debridement of infective foci, and valve repair when required. Operative mortality for tricuspid valve surgery in the setting of right‐sided IE is high with a range of 6%‐10%.^7^, ^8^

When the risk of cardiothoracic surgery in patients with tricuspid valve IE has been felt to be excessively high, less invasive percutaneous debulking procedures have been developed and offered to these high‐risk patients as a surrogate for open vegetectomy. This strategy looks to capitalize on the “inoculum effect”; an incompletely understood microbiologic phenomenon demonstrating attenuation of antibiotic therapy against high densities of bacteria in vitro.^9^ The predominant method has been with the utilization of percutaneous aspiration devices originally developed for the removal of intravascular materials, namely thrombi and emboli. Once such system, the AngioVac system, has been described in several case reports and case series dating from 2011 with positive implications for resolution or reduction in vegetation size, bacteremia, and clinical outcome.^10^

Experience with rheolytic thrombectomy catheters in the management of tricuspid valve IE has not been described to our knowledge. For our patient, the AngioJet rheolytic system was utilized successfully as a novel method for vegetation debulking. The AngioJet system is a mechanical thrombectomy tool studied in both peripheral vascular disease and coronary artery disease.^11^ It consists of a main unit with a high‐pressure infusion pump, which injects heparinized saline solution into a 5F dual lumen rapid exchange catheter. The saline solution exits at the catheter's tip distally through microholes and back into the collector lumen at high speeds, creating strong negative pressure (~−600 mm Hg), and hence, the mechanical force required for aspirating thrombus back down the catheter and into the collection system outside the patient.

No data exist on the use of rheolytic therapy in the management of endocarditis. One case report from Sulaiman et al^12^ describes the use of a mechanical thrombectomy catheter for the aspiration of an infected ileofemoral deep vein thrombosis as a means of source control with good effect. There are many reasons why this technique would be favorable in a subset of tricuspid valve IE patients with prohibitive surgical risk and failing standard medical therapy. The venous access required is relatively small in comparison with extracorporeal aspiration systems such as the AngioVac, and our patient was able to have this procedure performed with a single 14F venous sheath, although we could potentially have only used the 8.5F Agilis sheath. Anesthetic requirements for delivery of these rheolytic catheters are again, minimal, and of high significance in this subset of patients with unacceptably high surgical risk.

There are several limitations to this technique which must be considered. The AngioJet system was designed to be delivered intravascularly with an ability to direct side ports under fluoroscopy in an attempt to aspirate in a 360‐degree plane within the artery. To accurately direct the AngioJet catheter toward an open cavity is difficult and required simultaneous TOE guidance with the directable Agilis sheath. Even with this hybrid approach, a truly accurate representation of the catheters proximity to the vegetation was difficult to achieve and required multiple passes through the valve.

The immediate concerns with this technique and where it is limited in comparison with larger extracorporeal systems such as the AngioVac are the potential for large fragmentation of infective vegetation with septic emboli into the pulmonary vasculature and parenchyma, and the potential for large volume hemolysis and subsequent acute renal failure. Both these complications have the potential to lead life‐threatening sequelae including massive and submassive pulmonary emboli, pulmonary infarcts, pulmonary abscesses, and acute renal failure requiring dialysis. These are considered risks that are required to be taken, understanding the preclusions from open vegetectomy and the broader principles of the inoculum effect to increase antibiotic efficacy even with a partially aspirated or fragmented infective vegetation.

## 4 CONCLUSION

The use of an AngioJet rheolytic aspiration catheter was successful in the debulking of our patient's tricuspid valve vegetation and salvaging a near terminal presentation. This percutaneous technique appears reasonable to consider in patients with refractory TV endocarditis and prohibitive surgical risk as a means of vegetation debulking and infective source control.

## CONFLICT OF INTEREST

None declared.

## AUTHOR CONTRIBUTIONS

All authors contributed equally to this manuscript.

## ETHICAL APPROVAL

Relevant ethical approval for this study was obtained where required which included informed written consent for an anonymous case report from our patient.

## Supporting information

Video S1

Video S2

Video S3

Supplementary Material

## Data Availability

Data sharing is not applicable to this article, as no datasets were created or analyzed during the current study.
