# Supplementary material for: A case of percutaneous tricuspid valve infective endocarditis vegetation debulking using the AngioJet rheolytic catheter system—A novel therapeutic use
Source: Clin Case Rep. 2021 Jun 24;9(6):e04314. doi: 10.1002/ccr3.4314 (PMC8223887; doi:10.1002/ccr3.4314)
Supplement: Supplementary file 4 — Supplementary Material [file CCR3-9-e04314-s004.docx]

Supplementary Video 1 –

Biplane transoesophageal echocardiogram images from the gastric window demonstrating a large mobile echodensity on the anterior tricuspid valve leaflet consistent with an infective vegetation.

Supplementary Video 2 –

Fluoroscopy images demonstrating the AngioJet catheter passing from the end of the Agilis catheter across the tricuspid valve with transoesophageal echocardiogram guidance.

Supplementary Video 3 –

Biplane transoesophageal echocardiogram images from the mid-esophageal window demonstrating the AngioJet catheter passing from the end of the Agilis catheter across the tricuspid valve. Agitated saline exiting the distal AngioJet tip during aspiration attempts is visible.
